# Renal replacement therapy by the popliteal vein in a critical patient with COVID-19 in the prone position

**DOI:** 10.1590/2175-8239-JBN-2020-0190

**Published:** 2021-02-12

**Authors:** Rafael Gardone Guimarães, Arthur Pires Lacerda, Gabriela Portilho de Castro Rodrigues de Carvalho, Luiza Reis de Sales, Marina Perim Vásárhelyi, Matheus Pessanha Paixão

**Affiliations:** 1 Hospital dos Plantadores de Cana, Campos dos Goytacazes, RJ, Brasil.; 2 Faculdade de Medicina de Campos, Departamento de Medicina, Disciplina de Nefrologia, Campos dos Goytacazes, RJ, Brasil.

**Keywords:** Coronavirus Infections, Kidney, Renal Replacement Therapy, Popliteal Cyst, Pronation, Patients, Infecções por Coronavirus, Rim, Terapia de Substituição Renal, Veia poplítea, Pronação, Pacientes

## Abstract

This patient was a 73-year-old man who initially came to our service with acute respiratory failure secondary to COVID-19. Soon after hospitalization, he was submitted to orotracheal intubation and placed in the prone position to improve hypoxia, due to severe acute respiratory syndrome (SARS). On the third day of hospitalization, he developed acute oliguric kidney injury and volume overload. The nephrology service was activated to obtain deep venous access for renal replacement therapy (RRT). The patient could not be placed in the supine position due to significant hypoxemia. A 50-cm Permcath (MAHURKARTM, Covidien, Massachusetts, USA) was inserted through the left popliteal vein. This case report describes a possible challenging scenario that the interventional nephrologist may encounter when dealing with patients with COVID-19 with respiratory impairment in the prone position.

## Resumo

O paciente era um homem de 73 anos de idade que inicialmente veio ao nosso serviço com insuficiência respiratória aguda secundária à COVID-19. Logo após a internação, ele foi submetido à intubação orotraqueal e pronado para melhorar a hipóxia devido à síndrome respiratória aguda grave (SARS - do inglês "severe acute respiratory syndrome"). No terceiro dia de internação, o mesmo desenvolveu lesão renal aguda oligúrica e sobrecarga de volume. O serviço de nefrologia foi acionado para realizar acesso venoso profundo para terapia renal substitutiva (TRS). O paciente não pôde ser colocado na posição de decúbito dorsal devido a uma hipoxemia significativa. Um Permcath de 50 cm (MAHURKARTM, Covidien, Massachusetts, EUA) foi inserido através da veia poplítea esquerda. Este relato de caso descreve um possível cenário desafiador com o qual o nefrologista intervencionista pode se deparar ao lidar com pacientes com COVID-19 com problemas respiratórios e colocados em pronação.

## Introduction

We are experiencing the most important pandemic in recent history caused by a new coronavirus (SARS-CoV-2), with a significant impact on public health^1^^,^ 2. The virus is transmissible by droplets and contact^3^. Although the majority of cases are mild, approximately 5% of those infected develop severe acute respiratory syndrome (SARS), accompanied by acute kidney injury and multiple organ failure^4^. Many of these patients require central venous access for renal replacement therapy (RRT). The first option of vascular access in critical patients who need RRT is the internal jugular vein, while the second and third access options are the common femoral and subclavian veins, respectively. There are a number of barriers in obtaining vascular access for RRT in patients with SARS due to COVID-19, especially in those who develop significant hypoxemia and need to remain in the prone position to improve oxygenation. First, prone positioning eliminates access to the common femoral veins and subclavian veins and makes obtaining internal jugular venous access significantly more challenging, elevating the risk of iatrogenic pneumothorax. Second, the proximity of the internal jugular veins to the patient's airways increases the risk of viral contamination. Finally, many patients with COVID-19 will need prolonged treatment in the intensive care unit, increasing the risk of long-term central venous stenosis with dialysis catheters in the jugular or subclavian veins.

## Case report

This patient was a 73-year-old white man, hypertensive, who was admitted to the intensive care unit with acute respiratory failure arising due to COVID-19. Soon after hospitalization, he was submitted to orotracheal intubation and placed in the prone position to improve hypoxia, due to severe acute respiratory syndrome (SARS). On the third day of hospitalization, he developed oliguria, azotemia, and volume overload. The nephrology service was activated to obtain deep venous access for RRT. The patient could not be placed in the supine position due to significant hypoxemia. A long-term dialysis catheter was inserted through the left popliteal vein. He underwent conventional intermittent hemodialysis daily for 8 days. The blood flow during the sessions remained between 300 - 400 mL/min. Due to the severity of the clinical scenario, the patient ended up dying as a consequence of respiratory failure on the 11th day. This case report describes a challenging situation in obtaining vascular access when dealing with patients with COVID-19 in the prone position.

## Technical aspects

**Figure 1:**
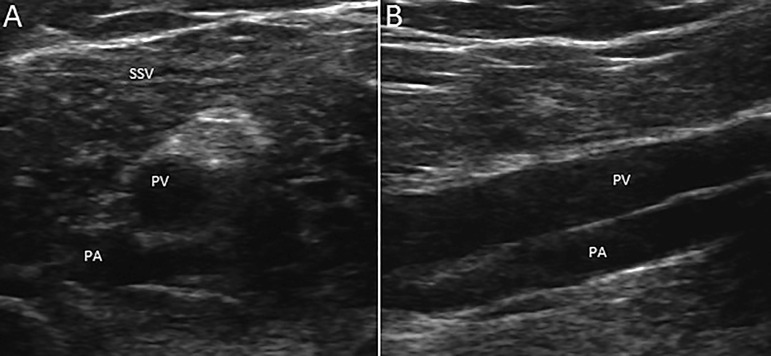
A and B. Venous duplex ultrasound B-mode images, axial and sagittal views. PA, Popliteal artery. PV, popliteal vein. SSV, small saphenous vein.

**Figure 2:**
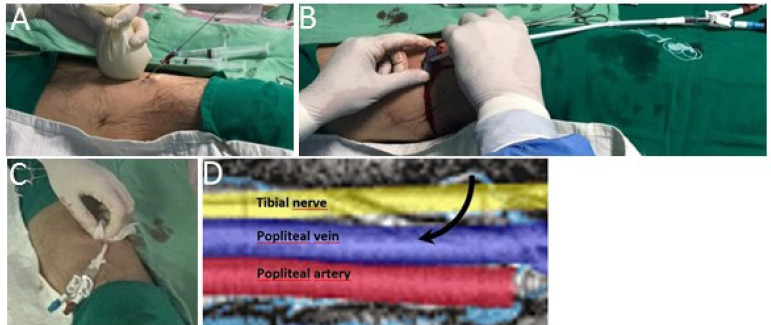
A-D. Accessing the popliteal vein in the popliteal fossa.

The left popliteal vein was evaluated by ultrasound (Figure 1). We used transversal and longitudinal sections to avoid any valve, being careful not to injure the superficially located tibial nerve or the popliteal artery, the deepest structure of the popliteal fossa. The popliteal vein was punctured using an 18GA x 7 cm needle, and a 0.035-inch guidewire was inserted without any resistance. A cut on the skin was made over the wire, the vein was dilated, and the 50-cm permcath was inserted with the aid of a 16F divisible introducer by the interventional nephrologist (Figure 2). The cuff was sutured in the subcutaneous tissue. Both arterial and venous routes were successfully tested and salinized. Conventional intermittent hemodialysis was started without difficulty.

## Discussion

In the literature, there is only one case report similar to the one already described^1^. In both cases, the patient was admitted with severe acute respiratory syndrome due to COVID-19 and required renal replacement therapy. He could not be positioned supine without immediately becoming hypoxic and decompensating. A long-term catheter was inserted through the popliteal vein. The advantages of using the popliteal vein for renal replacement therapy in critical patients with COVID-19 are substantial^1^. The popliteal vein is easily accessible in patients with SARS-CoV-2 in the prone position and in need of RRT^1^. In addition, it avoids access to the internal jugular veins and subclavian veins, thus reducing the risk of contamination and central venous stenosis, as these patients, for the most part, require prolonged hospitalization^1^. Regarding disadvantages, this access method is highly operator-dependent, requiring familiarization with the anatomy of the popliteal fossa and the use of an ultrasound device^1^. Another disadvantage is the risk of deep vein thrombosis, although this risk appears to be the same compared with catheters inserted in the jugular or subclavian veins^1^. In our limited experience, this complication did not occur. Finally, this method requires a longer dialysis catheter to reach the deep central vein, which could limit flow during RRT^1^. This unconventional choice of access was beneficial for both the patient and the team involved in his care. In view of this challenging scenario, we believe that the popliteal vein can and should be considered a good option for deep venous access for patients in this group who need RRT.
